# Reversal of Cardiac Electrical Heterogeneity Following Microsurgical Treatment of Cerebral Aneurysms: Longitudinal Changes in QTc and P-Wave Dispersion: A Retrospective Single-Center Study

**DOI:** 10.3390/jcm15134964

**Published:** 2026-06-25

**Authors:** Oguz Kaan Kaya, Veli Umut Turgut

**Affiliations:** 1 Department of Cardiology, Antalya Training and Research Hospital, University of Health Sciences, 07100 Antalya, Türkiye; 2 Department of Neurosurgery, Antalya City Hospital, 07080 Antalya, Türkiye; umutturgut07@gmail.com

**Keywords:** cerebral aneurysm, aneurysmal subarachnoid hemorrhage, QTc dispersion, P-wave dispersion, ventricular repolarization, atrial conduction heterogeneity

## Abstract

**Background:** Cerebral aneurysms and aneurysmal subarachnoid hemorrhage (aSAH) may induce cardiac electrical instability through autonomic dysregulation and an exaggerated neurohumoral stress response. Electrocardiographic (ECG) abnormalities, including QT/QTc prolongation, QTc dispersion, and P-wave dispersion, are recognized markers of ventricular repolarization heterogeneity and atrial conduction abnormalities associated with arrhythmogenic risk. However, data regarding the reversibility of these electrophysiological alterations following definitive aneurysm treatment remain limited. **Methods:** This retrospective, single-center study included 39 patients with cerebral aneurysms who underwent microsurgical clipping between January 2025 and May 2026 and 35 age- and sex-matched healthy controls. Standard 12-lead ECGs were evaluated at baseline (preoperative) and one month after surgery in the aneurysm group. QT interval, corrected QT (QTc) interval, QTc dispersion, and P-wave dispersion were assessed using standardized methods. Baseline transthoracic echocardiographic parameters, including left ventricular ejection fraction and left atrial diameter, were evaluated to minimize potential confounding related to structural cardiac abnormalities. Between-group and within-group comparisons were performed using appropriate statistical analyses. **Results:** Baseline demographic and echocardiographic characteristics were comparable between the aneurysm and control groups. Patients with cerebral aneurysms demonstrated significantly higher baseline QT interval, QTc interval, QTc dispersion, and P-wave dispersion compared with healthy controls. Following microsurgical treatment, significant reductions in QT interval, QTc interval, QTc dispersion, and P-wave dispersion were observed at one month compared with preoperative values, whereas PR interval and QRS duration remained unchanged. These findings suggest a partial normalization of cardiac electrical heterogeneity after definitive aneurysm treatment. **Conclusions:** Cerebral aneurysms are associated with increased ventricular repolarization and atrial conduction heterogeneity, reflecting autonomic-mediated cardiac electrical instability. The significant reduction in QTc dispersion and P-wave dispersion following microsurgical treatment suggests that these electrophysiological abnormalities may be at least partially reversible after aneurysm repair. ECG-derived markers such as QTc dispersion and P-wave dispersion may represent practical and non-invasive tools for monitoring cardiac electrical instability and recovery in patients with cerebral aneurysms.

## 1. Introduction

Aneurysmal subarachnoid hemorrhage (aSAH) and cerebral aneurysms are severe neurovascular conditions associated with substantial morbidity and mortality, requiring prompt diagnosis and multidisciplinary management. Contemporary guidelines emphasize that early aneurysm securing is a key determinant of neurological outcome and prevention of rebleeding [1,2,3]. In clinical practice, disease severity and treatment strategies are commonly guided by validated scoring systems, including the Hunt–Hess scale, the World Federation of Neurosurgical Societies (WFNS) classification, and the Fisher radiological grading system [4,5,6].

Beyond their primary neurological manifestations, cerebral aneurysms and aSAH may provoke a profound systemic stress response, particularly involving the cardiovascular system. Acute sympathetic overactivation and excessive catecholamine release have been implicated in myocardial injury, autonomic dysregulation, electrical instability, and the development of both atrial and ventricular arrhythmias [7,8,9,10]. This complex neurocardiac interaction is increasingly recognized within the framework of the *brain–heart axis*, which highlights the bidirectional interplay between acute neurological injury and cardiovascular dysfunction [11,12,13].

Electrocardiographic (ECG) abnormalities are frequently observed in patients with aSAH and include QT/QTc prolongation, ST–T wave abnormalities, conduction disturbances, and supraventricular or ventricular arrhythmias [14,15,16,17]. However, conventional ECG indices may not fully capture the extent of underlying electrical instability. Markers of ventricular repolarization heterogeneity, particularly QTc duration and QTc dispersion, have emerged as more sensitive indicators of arrhythmogenic substrate and adverse cardiovascular risk [18,19,20]. Likewise, P-wave dispersion has been recognized as a non-invasive marker of atrial conduction heterogeneity and susceptibility to atrial arrhythmias, particularly atrial fibrillation [21,22,23].

Although previous studies have documented electrocardiographic abnormalities in patients with aSAH, the temporal evolution of cardiac electrical heterogeneity following definitive aneurysm treatment remains incompletely understood. In particular, it remains unclear whether autonomic-mediated electrophysiological disturbances are reversible after successful microsurgical aneurysm treatment. Improved understanding of these dynamic electrophysiological changes may have important implications for perioperative cardiac monitoring and arrhythmic risk stratification in patients with cerebral aneurysms.

Therefore, the present study aimed to compare ECG-derived markers of cardiac electrical heterogeneity between patients with cerebral aneurysms and age- and sex-matched healthy controls and to investigate longitudinal changes in QTc dispersion and P-wave dispersion one month after successful microsurgical treatment.

## 2. Materials and Methods

### 2.1. Study Design and Setting

This retrospective, single-center study was conducted in collaboration with the Department of Neurosurgery at Antalya City Hospital, Antalya, Türkiye. The study was conducted in accordance with the Declaration of Helsinki and approved by the Ethics Committee of Antalya Training and Research Hospital, Antalya, Türkiye (Application No: 2027-283; Decision No: 10/14; approval date: 22 May 2026). Due to the retrospective nature of the study, the requirement for informed consent was waived.

### 2.2. Study Population

Eligible patients diagnosed with cerebral aneurysms who underwent microsurgical aneurysm clipping between January 2025 and May 2026 and fulfilled all predefined inclusion and exclusion criteria were retrospectively included in the study. The control group consisted of age- and sex-matched healthy individuals without known cardiovascular or neurological disease.

Exclusion criteria included a history of coronary artery disease, heart failure, significant valvular heart disease, reduced left ventricular systolic function, atrial fibrillation or other clinically significant arrhythmias, electrolyte imbalance, acute coronary syndrome, use of medications known to affect cardiac conduction (e.g., antiarrhythmic drugs), and inadequate ECG quality.

Clinical and neurosurgical characteristics of the aneurysm cohort, including rupture status, aneurysm location, Hunt–Hess grade, World Federation of Neurosurgical Societies (WFNS) score, Fisher grade, aneurysm size, and time to surgery, are summarized in Supplementary Table S1.

### 2.3. Echocardiographic Assessment

Baseline transthoracic echocardiographic examinations were performed to minimize potential confounding related to structural cardiac abnormalities. Left ventricular ejection fraction (LVEF) and left atrial anteroposterior diameter were recorded for all participants.

### 2.4. Electrocardiographic Analysis

Standard 12-lead electrocardiograms (ECGs) were recorded under resting conditions at a paper speed of 25 mm/s and a calibration of 10 mm/mV for all participants.

In the aneurysm group, ECG recordings were obtained at baseline (preoperative) and one month after microsurgical treatment. All ECGs were independently evaluated by two investigators blinded to clinical data, and disagreements were resolved by consensus to minimize measurement variability.

QT interval was measured from the onset of the QRS complex to the end of the T wave. Corrected QT (QTc) interval was calculated using Bazett’s formula. QTc dispersion was defined as the difference between the maximum and minimum QTc intervals measured across all measurable leads.

P-wave duration was measured from the onset to the end of the P wave, and P-wave dispersion was defined as the difference between the maximum and minimum P-wave durations across all measurable leads.

### 2.5. Statistical Analysis

Statistical analyses were performed using IBM SPSS Statistics for Windows, Version 26.0 (IBM Corp., Armonk, NY, USA). The distribution of continuous variables was assessed using the Kolmogorov–Smirnov test.

Normally distributed continuous variables are presented as mean ± standard deviation (SD), whereas non-normally distributed variables are expressed as median (interquartile range [IQR]). Categorical variables are presented as frequencies and percentages.

Between-group comparisons were performed using the independent-samples *t*-test or Mann–Whitney *U* test, as appropriate. Within-group comparisons were assessed using the paired *t*-test or Wilcoxon signed-rank test. Categorical variables were compared using the chi-square test or Fisher’s exact test, where appropriate.

A two-sided *p*-value < 0.05 was considered statistically significant.

A post hoc power analysis was performed using QTc dispersion, which represented one of the primary study endpoints. Based on the observed effect size (Cohen’s dz = 1.54), the achieved statistical power exceeded 99% at a two-sided alpha level of 0.05. The estimated minimum sample size required to achieve 80% statistical power was six patients, supporting the adequacy of the study sample size.

### 2.6. Use of Artificial Intelligence Tools

Artificial Intelligence (Ai)-Assisted Tools Were Used Solely for Language Refinement and Grammatical Editing During Manuscript Preparation. No Ai Tools Were Used for Data Analysis, Interpretation of Results, or Scientific Decision-Making. All Content Was Critically Reviewed and Approved by the Authors.

## 3. Results

### 3.1. Baseline Characteristics

A total of 39 patients with cerebral aneurysms who underwent microsurgical treatment and 35 age- and sex-matched healthy controls were included in the study. Neurosurgical characteristics of the aneurysm cohort are summarized in Supplementary Table S1. The cohort predominantly consisted of patients with ruptured aneurysms treated with microsurgical clipping.

Baseline demographic characteristics, including age, sex distribution, hypertension, and diabetes mellitus, were comparable between the aneurysm and control groups (Table 1). Similarly, baseline echocardiographic parameters, including left ventricular ejection fraction (LVEF) and left atrial anteroposterior diameter, did not differ significantly between the groups (Table 1).

### 3.2. Baseline Electrocardiographic Findings

At baseline, patients with cerebral aneurysms exhibited significantly higher QT interval, corrected QT (QTc) interval, QTc dispersion, and P-wave dispersion compared with healthy controls (Table 1). In contrast, no significant between-group differences were observed in PR interval or QRS duration.

These findings suggest increased ventricular repolarization and atrial conduction heterogeneity in patients with cerebral aneurysms prior to definitive surgical treatment.

### 3.3. Longitudinal Changes Following Microsurgical Treatment

Within-group analysis demonstrated significant reductions in QT interval and QTc interval at one month following microsurgical treatment compared with preoperative values (Table 2). Similarly, QTc dispersion and P-wave dispersion were significantly reduced during the postoperative period (Table 2). In contrast, no significant changes were observed in PR interval or QRS duration following surgery.

These findings indicate partial normalization of cardiac electrical heterogeneity after successful aneurysm treatment.

### 3.4. Graphical Analysis of Electrocardiographic Parameters

**Figure 1:**
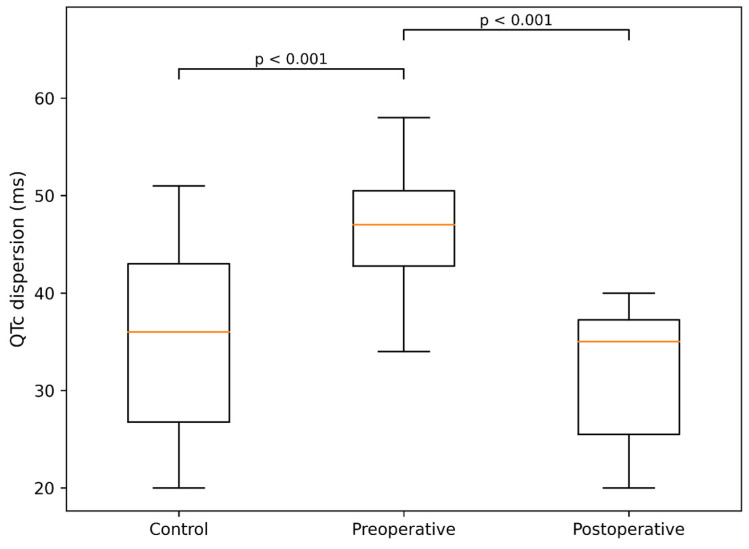
Changes in QTc Dispersion Before and After Microsurgical Treatment of Cerebral Aneurysms. Boxplots illustrating QTc dispersion values in healthy controls and in patients with cerebral aneurysms at baseline (preoperative) and one month after microsurgical treatment (postoperative). The central line represents the median, boxes indicate the interquartile range (IQR), and whiskers represent the range. QTc dispersion was elevated in the aneurysm group at baseline and decreased following microsurgical treatment.

**Figure 2:**
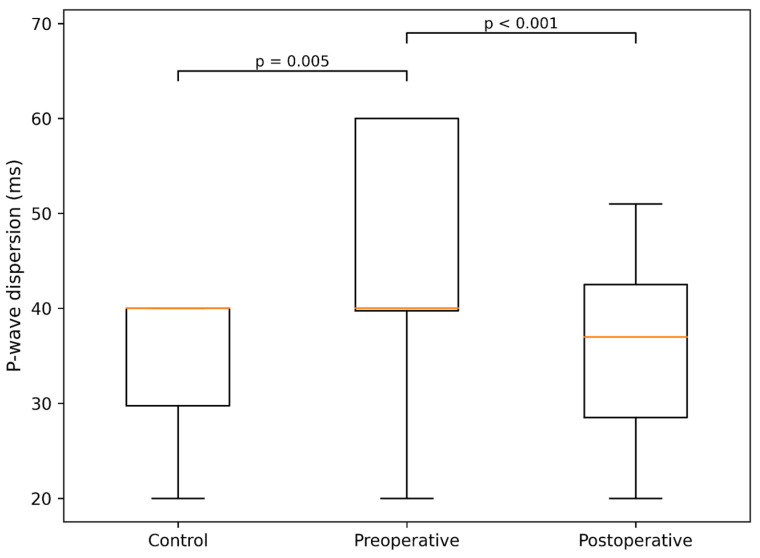
Changes in P-Wave Dispersion Before and After Microsurgical Treatment of Cerebral Aneurysms. Boxplots illustrating P-wave dispersion values in healthy controls and in patients with cerebral aneurysms at baseline (preoperative) and one month after microsurgical treatment (postoperative). The central line represents the median, boxes indicate the interquartile range (IQR), and whiskers represent the range. P-wave dispersion was elevated preoperatively and decreased one month after microsurgical treatment.

Graphical analysis demonstrated that QTc dispersion was highest at baseline in the aneurysm group and decreased significantly following microsurgical treatment (Figure 1). A similar pattern was observed for P-wave dispersion, which was elevated preoperatively and significantly reduced one month postoperatively (Figure 2).

## 4. Discussion

The principal finding of the present study is that patients with cerebral aneurysms exhibited significantly increased QTc dispersion and P-wave dispersion at baseline, both of which improved substantially one month after successful microsurgical treatment. These findings suggest that the cardiac electrical heterogeneity associated with cerebral aneurysms and aneurysmal subarachnoid hemorrhage (aSAH) may be at least partially reversible following definitive aneurysm treatment.

The cardiovascular consequences of aSAH are increasingly recognized within the framework of the *brain–heart axis*, whereby acute neurological injury triggers systemic autonomic dysregulation and excessive sympathetic activation [11,12,13]. Catecholamine surge has been implicated in myocardial injury, repolarization abnormalities, and electrical instability, predisposing patients to both atrial and ventricular arrhythmias [7,8,9,10]. Previous large-scale observational studies and landmark randomized trials have established the role of definitive aneurysm treatment in improving long-term outcomes and reducing the risks of rebleeding and aneurysm recurrence. These studies have also provided the evidence base for current treatment strategies comparing microsurgical clipping and endovascular coiling in patients with cerebral aneurysms [24,25,26,27,28].

Elevated catecholamine levels may impair myocardial membrane stability and increase ventricular repolarization heterogeneity, thereby contributing to QTc prolongation and increased QTc dispersion [29]. Similarly, autonomic imbalance may adversely influence atrial conduction properties, potentially explaining the increased P-wave dispersion observed in our cohort.

Previous studies have documented a broad spectrum of electrocardiographic abnormalities in patients with aSAH, including QT prolongation, ST–T wave abnormalities, and rhythm disturbances [14,15,16,17]. In addition, studies evaluating the impact of aneurysm treatment have reported partial normalization of ECG abnormalities following early intervention [30,31]. Our findings are consistent with these observations and extend the current literature by specifically demonstrating longitudinal improvement in both ventricular repolarization heterogeneity (QTc dispersion) and atrial conduction heterogeneity (P-wave dispersion) following microsurgical aneurysm treatment.

Emerging evidence also suggests that QTc prolongation following aneurysmal subarachnoid hemorrhage may have prognostic significance beyond arrhythmogenic risk. Zhang et al. reported that prolonged QTc intervals after aSAH were associated with worse neurological outcomes in patients undergoing either microsurgical clipping or endovascular embolization. These findings support the concept that electrocardiographic abnormalities may reflect not only cardiac electrical instability but also the severity of neurocardiac interaction following acute neurological injury. In this context, the observed reduction in QTc interval and QTc dispersion after successful aneurysm treatment in our study may represent a favorable electrophysiological response accompanying recovery from neurogenic stress.

These findings may also have mechanistic relevance in the context of neurocardiogenic syndromes such as neurogenic stunned myocardium and Takotsubo syndrome, in which transient myocardial dysfunction and electrocardiographic abnormalities commonly coexist [11,12,13,32,33]. Interestingly, normalization of ECG abnormalities in these conditions may lag behind clinical stabilization [34], supporting the plausibility of persistent yet potentially reversible electrophysiological alterations following acute neurological injury.

Although catecholamine levels were not directly measured in the present study, the observed reduction in QTc dispersion and P-wave dispersion following surgery may reflect attenuation of sympathetic overactivity and restoration of autonomic balance. Importantly, the absence of significant differences in baseline echocardiographic parameters between groups suggests that the observed electrocardiographic abnormalities were less likely to be attributable to underlying structural cardiac disease. Moreover, the predominance of ruptured aneurysms in this cohort may further support the contribution of acute neurogenic stress and autonomic dysregulation to the observed electrophysiological abnormalities.

From a clinical perspective, ECG-derived indices such as QTc dispersion and P-wave dispersion may serve as practical, inexpensive, and non-invasive tools for monitoring cardiac electrical instability and its resolution in patients with cerebral aneurysms. In clinical practice, these parameters may facilitate early identification of patients at increased arrhythmogenic risk and may complement perioperative cardiac surveillance and postoperative follow-up strategies.

Although QTc dispersion has been widely used as a surrogate marker of ventricular repolarization heterogeneity, its measurement remains subject to methodological limitations and interobserver variability, as highlighted by Malik and Batchvarov [19]. Nevertheless, QTc dispersion continues to be frequently employed in clinical research as a practical and non-invasive marker of electrical instability and arrhythmogenic risk.

Microsurgical clipping remains a well-established treatment strategy for both ruptured and selected unruptured cerebral aneurysms, providing durable aneurysm occlusion and a low risk of recurrence. In addition, microsurgical treatment may be considered in selected cases of recurrent or residual aneurysms following previous clipping or endovascular coiling, particularly when complete aneurysm exclusion cannot be achieved through endovascular techniques. Although the present study was not designed to compare different treatment modalities, the observed improvement in electrocardiographic parameters following successful aneurysm repair further supports the beneficial effects of definitive aneurysm treatment on the neurocardiac axis.

Importantly, our findings support the concept that the arrhythmogenic substrate associated with the *brain–heart axis* is dynamic and potentially reversible following definitive aneurysm treatment. Collectively, these findings underscore the importance of integrating cardiovascular assessment into the multidisciplinary management of patients with cerebral aneurysms.

## 5. Limitations

This study has several limitations. First, the retrospective and single-center design may introduce selection bias and limit causal inference and generalizability. Second, catecholamine levels were not measured, precluding direct biochemical confirmation of the proposed mechanisms underlying cardiac electrical instability. Third, arrhythmia outcomes were not systematically assessed using Holter monitoring or continuous telemetry; therefore, subclinical atrial or ventricular arrhythmias may have been underestimated.

Furthermore, serum electrolyte levels and cardiac biomarkers were not systematically available for all participants, and residual confounding related to biochemical factors influencing ventricular repolarization cannot be completely excluded. Although baseline echocardiographic assessment was performed to minimize confounding related to structural cardiac abnormalities, unmeasured cardiovascular factors may still have affected electrocardiographic findings. Finally, the absence of long-term follow-up precludes assessment of the persistence and prognostic significance of the observed electrocardiographic improvements.

Information regarding the use of medications with potential effects on ventricular repolarization, including QT-prolonging agents, before and after surgery was not systematically available because of the retrospective design. Therefore, the potential influence of pharmacological therapy on QT-related parameters cannot be completely excluded. In addition, the relatively small sample size may have reduced the statistical precision of our findings, although statistically significant differences were observed across several electrocardiographic parameters.

In addition, body mass index and ASA classification data were not consistently available for all participants and therefore could not be included in the present analysis.

Despite these limitations, the present study has several strengths, including longitudinal pre- and postoperative ECG assessment, blinded ECG analysis, and the inclusion of an age- and sex-matched control group. In addition, baseline echocardiographic evaluation helped minimize confounding related to structural heart disease. Future prospective, multicenter studies incorporating continuous rhythm monitoring, biochemical markers, and long-term follow-up are warranted to further validate these findings and clarify their clinical implications.

## 6. Conclusions

Patients with cerebral aneurysms exhibit increased ventricular repolarization and atrial conduction heterogeneity, reflected by elevated QTc dispersion and P-wave dispersion, suggesting autonomic-mediated cardiac electrical instability. The significant reduction in these electrocardiographic abnormalities following microsurgical treatment indicates that such electrophysiological alterations may be at least partially reversible after definitive aneurysm treatment.

QTc dispersion and P-wave dispersion may serve as practical, inexpensive, and non-invasive markers for monitoring cardiac electrical instability and recovery in patients with cerebral aneurysms. These findings support the concept of a dynamic and potentially reversible arrhythmogenic substrate within the *brain–heart axis* and highlight the potential role of ECG-derived markers in perioperative cardiac risk stratification and postoperative surveillance.

## Figures and Tables

**Table 1:** Baseline demographic, echocardiographic, and electrocardiographic characteristics of patients with cerebral aneurysms and controls.

Variable	Aneurysm (*n* = 39)	Control (*n* = 35)	*p* Value
Age, years	48 (43–52)	44 (41–49.5)	0.10
Male sex, *n* (%)	19 (48.7)	15 (42.9)	0.69
Hypertension, *n* (%)	10 (25%)	5 (14%)	0.25
Diabetes mellitus, *n* (%)	2 (5%)	2 (6%)	1.00
LVEF (%)	62 ± 4	63 ± 3	0.32
Left atrial anteroposterior diameter (mm)	34 ± 4	33 ± 3	0.28
PR interval (ms)	143.6 ± 23.7	143.6 ± 18.1	0.996
QRS duration (ms)	83 (74–90.5)	84 (80–92)	0.48
QT interval (ms)	385 (367.5–410)	368 (346–389)	0.004
Corrected QT (QTc, ms)	429 (420–450.5)	415 (402–436.5)	0.002
QTc dispersion (ms)	47 (42.8–51)	36 (26.5–43)	<0.001
P-wave dispersion (ms)	40 (40–60)	40 (30–40)	0.005

Data are presented as mean ± standard deviation or median (interquartile range), according to data distribution. LVEF: left ventricular ejection fraction; QTc: corrected QT interval.

**Table 2:** Longitudinal changes in electrocardiographic parameters before and one month after microsurgical treatment of cerebral aneurysms (*n* = 39).

Variable	Preoperative	Postoperative	*p* Value
PR interval (ms)	144.1 ± 24.3	144.4 ± 22.8	0.90
QRS duration (ms)	84 (76–92)	83 (74–90)	0.43
QT interval (ms)	391.7 ± 29.5	374.7 ± 31.0	0.021
Corrected QT (QTc, ms)	436.8 ± 22.3	417.5 ± 22.6	<0.001
QTc dispersion (ms)	48.5 ± 10.8	31.1 ± 7.6	<0.001
P-wave dispersion (ms)	40 (40–60)	37 (28–42.5)	<0.001

Data are presented as mean ± standard deviation or median (interquartile range), according to data distribution. QTc: corrected QT interval.

## Data Availability

The data supporting the findings of this study are available within the article and its Supplementary Materials. Additional data are available from the corresponding author upon reasonable request.

## Supplementary Materials

The following supporting information can be downloaded at: https://www.mdpi.com/article/10.3390/jcm15134964/s1, Table S1: Neurosurgical characteristics of patients with cerebral aneurysms undergoing microsurgical clipping (*n* = 39).
