# Supplementary material for: Reversal of Cardiac Electrical Heterogeneity Following Microsurgical Treatment of Cerebral Aneurysms: Longitudinal Changes in QTc and P-Wave Dispersion: A Retrospective Single-Center Study
Source: J Clin Med. 2026 Jun 25;15(13):4964. doi: 10.3390/jcm15134964 (PMC13362055; doi:10.3390/jcm15134964)
Supplement: Supplementary file 1 [file jcm-15-04964-s001.zip › jcm-4365162-supplementary.pdf]

Supplementary Table S1. Neurosurgical characteristics of patients with cerebral aneurysms undergoing microsurgical clipping (n = 39)

Variable	Value
**Ruptured aneurysm, n (%)**	31 (79.5)
**Unruptured aneurysm, n (%)**	8 (20.5)
**Hunt–Hess grade**	2 (1–3)
**WFNS score**	2 (1–3)
**Fisher grade**	3 (2–3)
**Aneurysm size (mm)**	6.8 ± 2.5
**Aneurysm location, n (%)**	
**– Middle cerebral artery (MCA)**	12 (30.8)
**– Anterior communicating artery (AComA)**	10 (25.6)
**– Internal carotid artery (ICA)**	9 (23.1)
**– Posterior communicating artery (PComA)**	5 (12.8)
**– Other**	3 (7.7)
**Microsurgical clipping**	39 (100)
**Time to surgery after admission (days)**	2 (1–4)

Data are presented as mean ± standard deviation, median (interquartile range), or n (%), as appropriate. WFNS: World Federation of Neurosurgical Societies; MCA: middle cerebral artery; AComA: anterior communicating artery; ICA: internal carotid artery; PComA: posterior communicating artery.
